# Board exam preparation resource trends in academic health sciences libraries serving colleges of osteopathic medicine programs

**DOI:** 10.5195/jmla.2026.2320

**Published:** 2026-07-01

**Authors:** Joanne Muellenbach, Kyle Robinson, Hsin-liang Chen, Harold S. Bright, Lori Fitterling

**Affiliations:** 1 jmuellenbach@chsu.edu, Director of Library Services, Health Sciences Library, California Health Sciences University, Clovis, CA; 2 krobinson@chsu.edu, Librarian Manager, Systems and Technical Services, Health Sciences Library, California Health Sciences University, Clovis, CA; 3 hsinliach@pcom.edu, Library Director, Library, Philadelphia College of Osteopathic Medicine, Philadelphia, PA; 4 hbright@atsu.edu, Director of Library Services, A.T. Still Memorial Library, A.T. Still University, Mesa, AZ; 5 LFitterling@kansascity.edu, Director of Library Services, Library, Kansas City University, Kansas City, MO

**Keywords:** Academic Health Sciences Libraries, Board Exam Preparation, Library Collection Development, Medical Libraries, Osteopathic Medicine, Undergraduate Medical Education

## Abstract

**Background:**

The study seeks to identify the board exam preparation (prep) resources made available to medical students by academic health sciences libraries and other departments within U.S. colleges of osteopathic medicine (COM) programs, and the extent to which these resources are utilized by medical students and educators.

**Methods:**

The study was conducted in two phases. In phase one, forty-two COM library directors were invited to complete a survey regarding their usage, funding, and access models for available board prep resources. In phase two, semi-structured interviews were conducted via Zoom to explore the library's role in providing access to these resources.

**Results:**

In phase one, thirty-five out of a possible forty-two survey respondents shared the top board exam prep resources to which their COM programs subscribed, as well as the top library resources that included board prep elements. In phase two, thirteen directors participated in Zoom interviews, which were recorded and transcribed to identify key trends in funding responsibility, resource selection, and librarian involvement. It was revealed that governance matters, digital format rules, partnerships drive impact, telling the data story is key, and one-stop guidance works.

**Conclusion:**

The two-phase study provided a comprehensive review of the specific resources that academic health sciences libraries and other departments offer for board prep within COM programs. Through analyses of survey and semi-structured interview data, this study identifies trends in resource availability and use, informing strategic planning for libraries, medical school administration, and library science education, ultimately enhancing student outcomes.

## INTRODUCTION

College of Osteopathic Medicine (COM) programs have been experiencing rapid growth within medical education. According to the American Association of Colleges of Osteopathic Medicine's (AACOM) website, approximately 38,000 future doctors of osteopathy (DO) physicians—nearly 30% of all U.S. medical students—were enrolled across the now 46 accredited COMs, which include 73 teaching locations in 36 states as of February 2026 [1]. Between 2015 and 2024, the reported 68,304 DO graduates reflect a 63.2% growth rate over the past decade [2]. In addition, 8814 new DO physicians were poised to begin residency training in July 2025, representing a 99.12% match rate [3].

Both osteopathic (DO) and allopathic (MD) medical programs share the goal of producing highly skilled physicians, and their curricula are increasingly similar. A study by Burch and colleagues found that both professions utilize standardized board exams covering the essential curriculum, noting that many DO students also take and pass the United States Medical Licensing Examination (USMLE) exams, underscoring the significant overlap in training [4]. Since 1997, osteopathic and allopathic programs have progressively converged pedagogically, particularly in preclinical and basic science education.

This convergence is further evidenced by the combined residency match process, which in 2020 transitioned to a single accreditation system that requires all residents and fellows to meet the same training standards [5]. Medical students pursuing either a DO or MD degree must graduate from accredited schools and pass national licensing exams to qualify for graduate medical education and independent practice. Performance on these exams—including the National Board of Osteopathic Medical Examiners (NBOME) Comprehensive Osteopathic Medical Licensing Examination (COMLEX) Levels 1 and 2 for DO students and the National Board of Medical Examiners (NBME) USMLE Steps 1 and 2 for MD students—is a critical factor in residency selection. Consequently, the unprecedented growth in board exam study resources is a direct response to the increasing importance of these licensing exams in medical training and career advancement.

Berry and colleagues reported a notable shift in students' use of study resources as they progress through the curriculum, with an increasing reliance on materials not provided by their academic institutions [6]. Supporting this trend, a systematic review and meta-analysis by Kann and colleagues evaluated 22 third-party resources and identified First Aid, UWorld, and Pathoma as the most frequently used by medical students preparing for board exams [7]. Yang and colleagues further explored factors driving this growing reliance on board prep tools, highlighting students' affinity for technology and online learning, perceived efficiency, and the adoption of pass-fail grading in many biomedical science courses as key contributors [8]. In a commentary, Matsko and Cervantes noted that the rise of third-party study tools, like flashcards and videos, often presents students with opportunities to use “shortcut” learning methods [9].

A survey of Swedish medical students by Bjurstrom and colleagues found that the most valued advantages of digital resources—including flashcards, videos, and artificial intelligence (AI)—were their availability, flexibility, and enhanced learning effectiveness compared to traditional methods [10]. Similarly, Makus and colleagues surveyed students at the University of Ottawa who created their own Anki decks (a spaced repetition flashcard system) and question banks, reporting that these non-traditional tools saved time by reducing the need to take extensive lecture notes and offered anytime access [11]. The survey also revealed that student developed Anki decks, alongside commercial medical education platforms like Osmosis, AMBOSS, and UpToDate, were among the most popular resources. Online communities further amplify this trend; forums such as r/medicalschool, r/step1, and r/medicalschoolanki on Reddit serve as platforms where students share study strategies for exams like the USMLE Step 1 [12]. At the time of writing, r/medicalschool alone boasted over 785,500 followers. While Reddit members could be anyone, including current or prospective medical students or faculty, this number far exceeds the number of U.S. allopathic and osteopathic medical graduates over the past decade, estimated at 260,000 [13, 14, 15]. These forums enable students to exchange and recommend extensive Anki decks, some containing more than 20,000 cards, and many begin their studies a year before their exam date.

Several studies have explored the impact of third-party board exam prep resources on academic performance. Hirumi et al. investigated the use of commercial off-the-shelf prep materials and found direct evidence that these resources improved students' performance on national licensing exams, particularly USMLE scores [16]. Similarly, Burk-Rafel and colleagues reported that students who engaged with multiple third-party resources and adopted effective study behaviors—such as beginning exam prep early, increasing the use of review books, and completing more practice questions—achieved higher Step 1 scores [17]. However, a study by Kortz and colleagues concluded that the number of board prep resources utilized, and the number of questions completed to prepare for COMLEX Level 1 were not related to a student's COMLEX Level 1 performance [18]. Kind and colleagues reported that students, when asked about the resources used to read and learn, rated question banks and board review books as the most useful for exam prep, and 80% ranked peers as the most influential in recommending resources. Hours spent reading for exams were the only significant predictor of USMLE Step 2 scores related to study habits [19]. Yet, Jeyaraju et al. cautioned that although methods such as spaced learning and frequent testing enhance learning efficiency over time, no single resource has been shown to provide a definitive competitive advantage on national licensing exams [20].

Medical educators and librarians have observed that many medical students are increasingly disengaging from their preclinical curriculum to focus on board exam prep, a shift that can negatively affect their finances. Ball and colleagues reported that, on average, students spend approximately $1,345 on test prep and third-party resources throughout their medical education [21]. While this amount may not seem excessive on its own, when combined with other unexpected expenses—such as clinical supplies and student fees—it can create significant financial stress, particularly for first-generation college students. Farhan and colleagues found that students often combine multiple third-party resources, such as First Aid and UWorld, to maximize their board exam performance, driven by studies showing these tools' effectiveness in improving USMLE Step 1 scores [22]. This reliance on multiple paid resources further exacerbates students' financial burden. Coupled with already overwhelming medical student debt, this situation risks deepening socioeconomic disparities within medical education.

Moreover, students are not only turning to paid resources for board prep. Mohri et al. reported that 87% of surveyed medical students used paid e-learning tools for their school exams, with 71.5% noting an improvement in their exam performance [23]. Many also made frequent use of free demo sessions to supplement their studies. Given this prevalence, it is crucial for medical education programs to carefully select and provide evidence-based board exam prep resources to support students effectively, reducing both academic pressure and financial strain.

Medical school administrators—especially those involved in academic affairs, student affairs, and health sciences libraries—should prioritize evaluating students' use of third-party resources and explore ways to enhance access to these tools. The results of such an evaluation could be particularly relevant to curriculum leaders, as they can inform the development and refinement of learning materials and guide more strategic allocation of resources within medical education programs. To best support students, medical schools should assess the available resources, recommend evidence-based options, integrate them into the curriculum, and explore subsidizing their cost to reduce financial barriers.

The extent to which librarians are embedded in the medical school curriculum and involved in curricular planning can significantly influence their participation in selecting and managing board exam prep resources. However, in many cases, these commercial off-the-shelf tools are produced by lesser-known publishers who market directly to individual students rather than to institutional libraries. A survey by Shultz and Berryman found that while many libraries licensed at least one of these “non-traditional” resources—such as UWorld, SketchyMedical, or Pathoma—high cost and the complexity of setting up individual user accounts posed significant barriers to broader adoption [24].

Within osteopathic medicine libraries specifically, the literature describing the provision of board exam prep resources has been limited. A 2024 study conducted by several of the current investigators documented board exam prep resources visible through COM library websites, providing a baseline snapshot of publicly listed offerings. However, website-based methods cannot fully capture how resources are funded and governed across units, how access is operationalized, which resources are being evaluated for renewal or cancellation, or how libraries engage in decision-making when funding is held outside the library [25]. To address this gap, we conducted a two-phase study combining a national survey of COM library directors with follow-up interviews. This approach was designed to move beyond “what is listed” toward understanding how resources are selected, funded, managed, evaluated, and communicated within institutional structures.

In this study, board exam prep resources are defined as institutionally provided materials intended primarily to support preparation for COMLEX and/or USMLE examinations, and, when applicable, COMAT (Comprehensive Osteopathic Medical Achievement Test) shelf-style examinations. These may include online question banks, video-based learning platforms, flashcard systems, analytics-enabled study tools, and review texts provided in electronic and/or print formats. Using both survey and semi-structured interview methods, the study aims to identify usage trends and provide actionable insights that can inform strategic planning for libraries, medical school leadership, and library science education. Ultimately, our goal is to support the development of more equitable, accessible, and effective learning environments that enhance student success. Given that the study's investigators are employed in health sciences libraries at COM institutions, our research focuses specifically on COM libraries and moves beyond identifying available board exam prep resources to achieve the following objectives:

- To identify the range of board exam prep resources made available to medical students by academic health sciences libraries and other departments within the U.S. COM programs.
- To determine the extent to which these resources are utilized by students and educators, and at what points within the curriculum they are accessed.
- To describe the library's role in providing access to and support for board exam prep resources, including governance, communication, and evaluation practices that shape librarian involvement.

## METHODS

### Participants

Invitations were distributed to the library directors of the 42 COM programs then accredited by AACOM in March 2025. This two-phase study included: (1) an online survey (phase one) and (2) semi-structured interviews (phase two). The Institutional Review Board (IRB) at the first author's institution classified the study as exempt and not human subjects research (IRB #2025-006, California Health Sciences University).

### Phase One: Online Survey

A 10-item survey and a separate demographic questionnaire were developed in Qualtrics (Supplemental Appendix A - B). Survey items captured: (1) which board prep resources were available, (2) which unit funded each resource (library vs non-library), (3) access models, and (4) renewal/cancellation considerations. An initial invitation with the survey link was sent to the participants. They were sent a reminder after two weeks, followed by a final reminder. Between March and April 2025, 35 participants completed the survey, yielding an 83% response rate.

To reduce respondent burden and minimize misclassification, resource-funding questions were split into two parallel items: Q1a asked respondents to select resources supported by the library, and Q2a asked respondents to select resources supported by non-library units. This structure allowed respondents to report the same resource under the appropriate funding source at their institution without requiring repeated per-resource branching logic.

The instrument underwent expert review by eight osteopathic health sciences librarians to assess clarity, completeness, and alignment with study objectives (face and content validity); this feedback informed revisions prior to distribution.

### Phase Two: Semi-Structured Interviews

Survey respondents could volunteer for a follow-up interview. Semi-structured Zoom interviews (~30 minutes) were conducted using an interview guide (Supplemental Appendix C) to explore governance and decision-making, evaluation practices (e.g., usage metrics and stakeholder feedback), library roles and contributions, and operational challenges. Interviews were recorded with permission and transcribed for analysis. Thirteen out of 35 survey respondents participated in the online interviews in April and May 2025.

### Data Analysis

The investigators used Excel to calculate frequencies and percentages and applied descriptive statistics to analyze the distribution of resources and funding. The qualitative analysis followed a systematic, multi-step process: two research team members independently reviewed transcripts to generate initial codes, and any discrepancies were reconciled through discussion to establish a shared codebook. Given the exploratory nature of this study, intercoder reliability tests were not conducted. These codes were subsequently grouped into themes aligned with the phase two research questions—specifically, library roles, budget operations, contributions, and challenges. Finally, representative quotations were selected to exemplify these themes while strictly preserving participant anonymity.

## RESULTS

Overall, the study achieved strong participation: 35 of 42 library directors (83%) completed the phase one survey, and 13 directors participated in the phase two semi-structured interviews.

### Phase One – Online Survey

#### Respondent and Program Characteristics.

Thirty-five of the 42 invited COM library directors completed the phase one survey (83%). Because all survey items were optional, denominators vary across questions, and item-level response counts are reported in Table 1. Respondents' years serving as academic health sciences library directors (n=34) ranged from 1 to over 16 years, with most reporting 1-5 years (44%). The number of DO students served (n=34) most commonly fell between 501-1000 students (50%), with fewer serving more than 1000 (35%) and fewer serving fewer than 500 (15%). For institutional resource characteristics, respondents’ annual e-resource budgets (n=31) ranged widely, from $0 to $250K (3%) to more than $1M (39%). In contrast, annual print-resource budgets (n=31) were generally modest, with the majority reporting $0-49K (87%).

**Table 1:** Respondent and Program Characteristics (N=35)

Characteristics	N	%
Years as Library Director (n=34)	• 1-5 years: 15	44.1%
	• 6-10 years: 7	20.6%
	• 11-15 years: 7	20.6%
	• 16+ years: 5	14.7%
Approximate Number of DO Students Served (n=34)	• <500: 5	14.7%
	• 501-1,000: 17	50.9%
	• >1,000: 12	35.3%
Annual E-resource Budget (n=31)	• $0-250K: 1	3.2%
	• $250-500K: 8	25.8%
	• $500K-750K: 4	12.9%
	• $750K-1M: 6	19.4%
	• Over $1M: 12	38.7%
Annual Print Resource Budget Band (n=31)	• $0-49K: 27	87%
	• $50K-99K: 3	10%
	• Over $100K: 1	3%

#### RQ1. What board exam prep resources are financially supported by the libraries?

All responding COM libraries provided at least one dedicated board prep resource. The most common library-supported subscriptions included First Aid Forward, Boards and Beyond, TrueLearn (COMLEX 1 & 2), and Osmosis (Table 2). Some libraries also continued to support older or supplemental question banks such as BoardVitals and ExamMaster, although these were less common. In addition, libraries frequently provide access to review books (print and e-book) and multimedia study aids such as Osmosis, AMBOSS, SketchyMedical, or OnlineMedEd, supplementing the question banks with video lectures, imaging modules, or reference content. Supplemental Appendix D includes a complete listing of the board exam prep resources mentioned in our study, along with brief descriptions.

**Table 2:** Board Exam Prep Resources Funded by Libraries and Other COM Departments (n = 35)

Resource	Library Subscribes	Other Department Subscribes	Total
TrueLearn - COMLEX Level 1	3	20	23
TrueLearn - COMLEX Level 2	3	19	22
ScholarRx USMLE Study Tools	1	8	9
TrueLearn - COMAT Shelf Exams	2	6	8
OnlineMedEd	0	7	7
Osmosis	3	4	7
Boards and Beyond	4	3	7
AMBOSS	1	5	6
TrueLearn - COMLEX Level 3	1	5	6
UWorld - COMLEX Level 1	1	5	6
UWorld - COMLEX Level 2	1	5	6
First Aid Forward	5	1	6
UWorld - USMLE Step 1	1	4	5
UWorld - USMLE Step 2	1	4	5
Pathoma USMLE Step 1 and Medical Course Review	2	3	5
Kaplan - COMLEX Level 1 + Step 1 Qbank	0	4	4
Blueprint/Med School Tutors	0	1	1
Kaplan - COMLEX-Level 2 + Step 2 Qbank	0	1	1

**Caption:** This table summarizes survey responses from 35 COM library directors, showing which board exam prep resources are funded by libraries and which are supported by other departments (e.g., Dean's offices, Academic Affairs). Respondents could select multiple resources.

#### RQ2. What board exam prep resources are financially supported by other (non-library) COM departments?

Survey responses revealed that high-cost subscriptions, including TrueLearn (COMLEX Level 1, Level 2, and COMAT Shelf Exams), ScholarRx, and OnlineMedEd, were often funded by non-library units such as the Dean's office, Academic Affairs, or student support departments (Table 2). Libraries, in contrast, typically focused their budgets on lower-cost resources such as print or e-book review collections and assumed responsibility for the technical support of institutionally funded tools.

#### RQ3. What additional board exam prep resources are available through the libraries or other COM departments?

Beyond core question banks, the COM library directors reported providing supplemental or legacy resources, such as BoardVitals or the Lippincott, Williams and Wilkins (LWW) Board Review Series, to cover specific exam needs. Multimedia platforms (e.g., Draw It To Know It, Osmosis, Sketchy) were also cited, typically purchased by either libraries or academic departments to complement core Q-banks (Table 3). These findings show that COMs often assembled tool portfolios to meet diverse student preferences and curricular requirements.

**Table 3:** Supplemental and Legacy Board Exam Prep Resources Provided by COM Programs (N=35)

Resource	No.
BoardVitals	5
Board Review Series (LWW)	3
StatPearls	2
ExamMaster	2
COMSAE (NBOME)	2
COMQUEST Level 1 and 2	2
First Aid Print Books	2
Case Files	2
Step-Up to Medicine (LWW)	2
Test-Taking Strategies Session (Dr. Eftekar @ Northwestern Medical Review)	1
Draw It To Know It	1
Blueprints e-books	1
Test Prep for the USMLE: Pathology Q&A (Thieme)	1
Lecturio	1
MedOne Adaptive Learner (Thieme)	1

**Caption:** This table lists additional or legacy board exam prep resources made available by COM libraries or other departments, including review series, multimedia tools, and institution-specific offerings that complement core subscriptions. Respondents could select multiple resources.

#### RQ4. Which board exam prep resources are being considered for new subscriptions, or for cancellation, and why?

Library directors reported that rising subscription costs were the primary factor prompting consideration of cancellations or substitutions. For example, several COMs had discontinued or planned to discontinue expensive tools such as UWorld or First Aid Forward, replacing them with more affordable alternatives or bundled packages. To manage costs, institutions also pursued consortia purchasing agreements or multi-year licenses to secure discounts. Renewal decisions were frequently tied to usage analytics (logins, number of questions completed, account activations) and student demand. Tools with high usage or strong student support were prioritized for renewal, while those with low engagement were flagged for cancellation.

### Phase Two – Online Semi-Structured Interviews

Thirteen library directors (all phase one respondents who volunteered for follow-up) participated in 30-minute Zoom interviews during April and May 2025. The sessions provided a deeper context on institutional processes, library roles, and challenges related to board exam prep resources. The findings are summarized below, focusing on four phase two research questions.

#### RQ1. What is the role of the library in the management of board exam prep resources?

Participants described a wide spectrum of library involvement, ranging from central negotiator to supporting facilitator. In some COMs, the library led resource selection, licensing, and vendor management, actively advocating for student access. In others, the library's role was limited to troubleshooting access or providing information, while departments such as academic affairs made purchasing decisions. As one director explained, “our library is basically the go-between for students and vendors. We handle the contracts and make sure everything runs smoothly.” Another library director contrasted this with, ”we don't decide what to buy; the dean's office tells us what they've budgeted for, and we just set up access." These examples illustrate how governance structures strongly influence the extent of the library's responsibility.

#### RQ2. What impact do board exam prep resources have on the library budget and operations?

Directors emphasized that board prep resources often represent a significant financial burden, especially for those libraries with smaller budgets. Rising subscription costs forced institutions to consider cancellations, substitutions, or shared purchasing. Several directors described seeking consortia or multi-year agreements to reduce costs, while others relied on co-funding models with academic departments. Even when not the budget holder, libraries invested time and staff resources in managing licenses and access, and in providing student support. Financial pressures frequently shaped how libraries allocated resources and prioritized services, underscoring the operational costs of sustaining these tools.

#### RQ3. What contributions related to board exam prep resources has the library provided to COM programs?

Libraries consistently contributed expertise in licensing, vendor relations, and, in particular, the analysis and reporting of usage analytics, which directors used to advocate for renewals. A number of libraries have also developed student-facing supports, such as LibGuides, resource portals, or “board prep boot camps,” that consolidate all available resources in one place, regardless of whether they are funded by the library or another unit. These initiatives were widely credited with raising student awareness and boosting engagement. One director noted, “we created an online guide so students can easily find all the board exam prep tools we offer. It's now one of our most visited pages.” Collectively, these contributions positioned libraries as essential facilitators of student support, even when they were not the primary funders.

#### RQ4. What challenges related to board exam prep resources are COM libraries facing?

The most prominent challenges were governance and sustainability. Directors stressed that having formal representation on curriculum or budget committees was crucial to ensuring alignment between student needs and resource decisions. As one director put it, “when we have a seat at the table during budget discussions, renewals for these resources are much smoother.” In contrast, those excluded from governance described learning about cancellations or new purchases after decisions were made, leaving them reactive and less able to advocate with evidence. Participants also cited the need for stronger cross-department partnerships, such as advisory task forces or joint planning with academic affairs departments, which helped turn annual budget battles into collaborative processes. Finally, several directors raised AI as an emerging challenge. While students may experiment with AI tools, institutions remain cautious. Libraries may also hesitate to adopt limited library-specific AI-enhanced board exam prep products or third-party solutions due to institutional AI policies still under development, concerns about AI accuracy and hallucination, significant professional privacy considerations, and vendor clauses that restrict how AI may consume or reuse licensed content.

### Cross-Phase Data Synthesis from Phase One and Phase Two Results

The integration of phase one survey results and phase two semi-structured interview findings revealed several recurring themes that describe how COM libraries support board exam prep. Table 3 summarizes these themes, pairing quantitative evidence from the phase one survey with qualitative insights from the phase two interviews to highlight consistent patterns and notable differences across the two phases.

## DISCUSSION

Our findings indicate that COM libraries play a central role in supporting board exam preparation. Even though high-cost tools can be funded by other campus units, libraries remain essential by providing the expertise and direct student support these resources require. Subscription costs, usage data, and governance structures heavily shape renewal and cancellation decisions, and directors reported additional challenges, including tight budgets, uneven communication, and the still-evolving role of generative AI in exam prep. Based on the survey results from phase one and the interview insights from phase two, Table 4 depicts several patterns and a few notable variances in board exam prep resources across the COM programs.

**Table 4:** Integrated Themes from Phase One and Phase Two on COM Library Support for Board Exam Prep

Theme	Evidence from phase one (survey)	Evidence from phase two (semi-structured interviews)
Board Exam Prep as a Universal Priority	100% of respondents reported providing at least one board exam prep resource to students.	Directors emphasized board exam prep as “essential” for COMLEX/USMLE success.
Divergent Funding Models and Convergent Expertise	~50% of high-cost resources are funded by non-library units; others are funded by library budgets.	Libraries contribute evaluation, licensing, and management expertise regardless of funding source.
Cost–Evidence Decision Cycle	Cost pressures prompted cancellations or substitutions; usage analytics are commonly used for renewals.	Usage data and student feedback drive retention; low engagement prompts cancellation or replacement.
Impact of Governance and Communication	Libraries with joint funding/governance reported fewer funding issues.	Inclusive governance is linked to proactive planning; siloed decision-making causes inefficiencies.
Generative AI (GAI) on the Horizon	Not assessed in the survey.	GAI in board exam prep is discussed extensively; institutions are in early, exploratory stages of policy development.

**Caption:** This table integrates survey and focus group data to identify recurring themes in COM library support for board exam prep resources, including institutional priorities, funding models, governance, and the emerging role of AI.

### Alignment with Prior Literature

Previously cited studies have documented a shift toward third-party resources as students advance through medical curricula, with frequent use of First Aid, UWorld, and Pathoma, and with strong preferences for videos, flashcards, and other formats [6–9]. Our results support that body of work, as all responding COM libraries provided at least one dedicated board exam prep resource, and many supported multiple resources that mirror popular national categories, question banks, concise review texts, and multimedia platforms such as Osmosis, AMBOSS, Boards and Beyond, and Sketchy.

Consistent with evidence that students value availability, flexibility, and perceived efficiency [10, 11], libraries reported developing LibGuides, portals, and boot camps to aggregate access and instruction, helping to support peer-to-peer recommendations in online communities [12–15]. Overall, the institutional snapshot from our study reflects the student demand trends identified in earlier research, while also clarifying which units fund various tools and how access is coordinated across campuses.

This study also builds on earlier descriptions of board exam prep resources available in U.S. COM libraries by offering mixed-methods evidence about how these resources are funded, governed, implemented, and assessed. Whereas Muellenbach et al. [24] documented resources visible through COM library websites, website-based analyses cannot fully reveal cross-unit funding measures, account workflows, decision-making authority, or the extent of library involvement when subscriptions originate outside the library. This study fills that gap.

### Institutional Priority and Evolving Library Roles

Both phase one and phase two data confirm that board exam prep support is an institutional priority across osteopathic programs. The initial survey highlighted high subscription costs for board resources, which were further reinforced in interviews, where library directors and deans alike stressed the critical importance of student success on COMLEX and USMLE exams. This shared understanding underscores that providing these tools at the university level is seen as non-negotiable.

While most stakeholders expressed commitment, the extent of the library's commitment varies. Phase one quantitatively showed that about half of the funding for these resources originates outside the library budget, suggesting a limited library role in those cases. Phase two data expanded on this, detailing how some libraries are leaders in resource access/delivery, while others remain more peripheral. Both phases emphasize that libraries bring expertise to the discussion, particularly in managing access, analyzing usage, and deploying resources. Even when libraries are not budget holders, their expertise is often relied upon to ensure effective resource utilization. Taken as a whole, the findings point to an opportunity for institutions to align governance, budgeting, and library expertise to strengthen student-centered outcomes.

### Financial Pressures and Student-Driven Decisions

The issue of funding consistently occurred as a challenge across both phases of this study. Phase one data indicated reliance on non-library funds and difficult choices driven by cost. Phase two interviews provided anecdotal evidence, with directors recounting tough negotiations and instances in which high-cost resources, such as UWorld and Boards and Beyond, had to be dropped. This shows a clear pattern: budget constraints are common, leading to a consistent trend of seeking cost-effectiveness through consortia deals, cheaper alternatives, or limiting duplicative resources (print and digital). While phase one quantified funding sources, phase two offered deeper insights into the internal processes and “politics” of budget approvals, presenting a picture of the financial constraints involved.

Both phases' results strongly affirm a student-centered approach, in which students' needs and behaviors during board study drive resource decisions. The high adoption rates observed in the survey reflect student demand, a point detailed in interviews that described how student feedback (via committees or direct requests) and heavy usage patterns directly contribute to resource acquisition and renewal. Another insight is the critical importance of raising student awareness of available resources, often achieved through online guides, such as LibGuides, classes, and workshops. These findings illustrate how student engagement not only shapes resource selection but also remains essential to ensuring those resources are effectively discovered and used.

### Governance and the Library's Role

The benefits of strong communication and collaborative governance between the library and other stakeholders are a clear theme in this study. Survey responses indicate joint funding or cross-departmental support, while the interview testimonies about how partnerships “drive impact.” Libraries that operate independently in acquiring board-prep resources often face challenges that lead to reactive rather than strategic management. Conversely, inclusive governance, where libraries are embedded in the decision-making process, results in more coherent and sustainable board prep support and a more streamlined user experience.

### Generative AI and Emerging Tools

Finally, the topic of AI within board exam prep resources represents a new divergence between the two phases. Phase one focused on existing resource subscriptions and did not note AI's emergence because of the timeframe. However, phase two interviews revealed that AI is very much on the minds of library directors and administrators. Institutions are cautiously exploring AI tools and developing policies, while also being aware of students' unofficial use. As these tools mature, they may influence how students interact with question banks, adaptive learning systems, and other exam preparation platforms. This suggests potential shifts in future resource offerings and highlights a need for ongoing monitoring and research in this area.

### Limitations

This study has several limitations. First, while survey and interview responses offered valuable insights, they relied on self-reported information from the library directors. Although many participants consulted institutional records or contacted other departments to verify details, the data may still reflect institutional perspectives rather than objective usage metrics. Second, the study focused exclusively on colleges of osteopathic medicine, which may limit the generalizability of the findings to allopathic medical schools or other health professions programs. Finally, the study primarily examined institutionally licensed board exam prep resources. It did not capture the full range of student-driven or informal resources – such as peer-recommended free tools or individually purchased subscriptions – which may also significantly contribute to board exam preparation.

### Future Research

Future research should build on this director-level baseline by examining the broader board exam prep ecosystem across COM programs. First, studies should investigate cross-departmental funding and decision-making to clarify which administrative units (e.g., Academic Affairs, Dean's Offices, Student Success) bear the financial responsibility for high-cost resources, where resource requests originate, what criteria are used for selection and renewal, and how these subscriptions are incorporated into long-term budget planning. Second, researchers should directly engage students—the primary end-users—through surveys and focus groups to assess satisfaction, perceived learning curves for specific platforms, and whether institutionally provided tools are viewed as more “efficient” or valuable than personally purchased subscriptions, alongside relevant indicators of learning and performance where feasible. Finally, research should include college leadership perspectives to understand how board prep support fits within institutional strategy, including how resource investments align with the COM mission and accreditation expectations, and how leaders conceptualize board prep support in relation to COMLEX and USMLE performance goals.

## CONCLUSION

This study provides information on the role of medical libraries in COMs supporting a key success metric in medical education, COMLEX and USMLE board scores, through the provision of board exam prep resources. All COMs surveyed identified at least one resource for COMLEX or USMLE board prep. We identified the board exam prep resources made available at COM institutions, with TrueLearn COMLEX 1 being the most common. Results show that while such resources are considered critical for student board exam success, funding models among COM libraries and other departments are highly diverse. The survey highlighted that funding for expensive resources often comes from outside the library, with COM academic affairs and Dean's offices often funding and making board exam prep product choices.

During the semi-structured interviews, respondents revealed that libraries, regardless of their role in the budgetary process, are essential contributors to the success of resource deployment. Librarian expertise in licensing resources, vendor relations, troubleshooting, and managing resource access is considered key, positioning librarians as essential partners in the acquisition and use of board exam prep materials.

One important theme that emerged from this two-phase study was the strong link between the library's level of shared decision-making for these resources and students' perceived success in using them. Libraries with more formal input on the COM curriculum or budget mechanisms reported better communication regarding decision-making, closer alignment with COM needs and outcomes, and a more proactive approach to resource management. In contrast, libraries without this input faced less alignment with the COMs they served and more reactive approaches to resource acquisition, highlighting the necessity of intra-departmental university partnerships and productive communication channels.

The study also underscores the student-driven nature of resource selection. High student usage and positive feedback are primary drivers of resource renewal and acquisition. This dynamic creates a feedback loop where student demand informs acquisitions, usage data confirms value, and this evidence is used to advocate for continued funding. The emergence of AI in board exam prep, while not captured in the survey, was a significant topic in the phase two interviews, suggesting that this will be a crucial area for future research and resource strategy.

This important baseline information can be used by COM school administration, especially within the academic affairs and health sciences library departments, to help identify trends across U.S. osteopathic medical schools to justify the need for additional resources and services. These results will also assist university administration and library leadership in collections budget planning for their current and future academic health sciences libraries and COM programs.

## Data Availability

Data supporting the study's findings are available in the Supplementary Information. The data is also available through the Open Science Framework (OSF) at https://osf.io/8pm4v/overview.

## SUPPLEMENTAL FILES

**Appendix A:** Phase One Survey Questions

**Appendix B:** Phase One Survey Demographic Questions

**Appendix C:** Phase Two Interview Questions

**Appendix D:** Master List of Board Exam Prep Resources and Brief Descriptions

## References

[1] About AACOM. American Association of Colleges of Osteopathic Medicine (AACOM) [Internet]. 26 Aug 2025 [cited 12 Feb 2026]. https://www.aacom.org/about-us.

[2] Ali A. Osteopathic medical college graduates by gender 2000-2024. American Association of Colleges of Osteopathic Medicine (AACOM) [Internet]. 2 Sep 2025 [cited 12 Feb 2026]. https://www.aacom.org/searches/reports/report/osteopathic-medical-college-graduates-by-gender-2000-2024.

[3] Guercio E. Report on osteopathic medical school GME placements in 2025 matches. American Association of Colleges of Osteopathic Medicine (AACOM) [Internet]. 27 May 2025 [cited 12 Feb 2026]. https://www.aacom.org/searches/reports/report/report-on-osteopathic-medical-school-gme-placements-in-2024-matches.

[4] Burch J, Leavitt R, Smith F, Curtis JP. Common paths in medical education: an updated look at the training of allopathic, osteopathic, and naturopathic physicians. Integr Med (Encinitas). 2022 Nov;21(5):20–29. PMID: 36643213; PMCID: PMC9831133. PMC9831133 36643213

[5] AOA Media Team. AOA, ACGME and AACOM usher in new era of single accreditation for graduate medical education. American Osteopathic Association (AOA) [Internet]. 30 Jun 2020 [cited 12 Feb 2026]. https://osteopathic.org/2020/06/30/aoa-acgme-and-aacom-usher-in-new-era-of-single-accreditation-for-graduate-medical-education-2/.

[6] Berry A, Campbell A, Li D, Bay C, Ikonne U. An Investigation of second-year medical students’ use of outside resources at two institutions. Med Sci Ed. 2025. 35:847–862. 10.1007/s40670-024-02243-1 PMC12058637 40353005

[7] Kann MR, Huang GW, Pugazenthi S, Kann R, Chen D, Hardi A, Zehnder N. Unlocking medical student success: A systematic review and meta-analysis of third-party resources used for medical education and USMLE board preparation. Med Sci Educ. 2024;34(6):1603–1622. 39758474 10.1007/s40670-024-02116-7 PMC11699012

[8] Yang W, Quesnelle KM, Porter-Stransky A. Learning together: a narrative review of external resources for medical education through a shared student-faculty lens. Ann Med. 2025;57(1):2483971. 10.1080/07853890.2025.2483971 40152754 PMC11956101

[9] Matsko C, Cervantes J. Third-part resources: The new wave of medical education. Med Sci Educ. 2025. 35:1089–1092. 10.1007/s40670-024-02262-y 40353024 PMC12058618

[10] Bjurstrom MF, Lundkvist E, Sturesson LW, Borgquist O, Lunden R, Fonsson Fagerlund M, Lipcsey M, Kander T. Digital learning resource use among Swedish medical students: insights from a nationwide survey. BMC Med Educ. 2025;25:1–14. https://doe.org/10.1186/s12909-07446-7 40500719 10.1186/s12909-025-07446-7 PMC12153187

[11] Makus D, Kashyap A, Labib M, Humphrey-Murto S. A curriculum ignored? The usage of unofficial commercial and peer learning resources in undergraduate medical education at a Canadian medical school. Med Sci Educ. 2023;33(6);1379-1388. Doi: 10.1007/s40670-023-01899-5. 38188389 PMC10767013

[12] Ronner, Lukas; Linkowski, Lauren EdD. Online forums and the “step 1 climate”: perspectives from a medical student Reddit user. Acad Med. 2020 Sep;95(9):1329–1331. | DOI: 10.1097/ACM.0000000000003220. 32079938

[13] Ali A. Osteopathic medical college graduates by race/ethnicity 2000-2024. American Association of Colleges of Osteopathic Medicine (AACOM) [Internet]. 3 Sep 2025 [cited 12 Feb 2026]. https://www.aacom.org/searches/reports/report/osteopathic-medical-college-graduates-by-race-ethnicity-2000-2024.

[14] 2024 FACTS: enrollment, graduates, and MD-PhD data by institution. B-2.1 Total graduates by U.S. medical school, gender, and year, 2015-2016 through 2019-2020. Association of American Medical Colleges (AAMC) [Internet]. [cited 12 Feb 2026]. https://www.aamc.org/data/facts.

[15] 2024 FACTS: enrollment, graduates, and MD-PhD data by institution. B-2.2 total graduates by U.S. medical school, gender, and year, 2020-2021 through 2024-2025. Association of American Medical Colleges (AAMC) [Internet]. [cited 12 Feb 2026]. https://www.aamc.org/data/facts.

[16] Hirumi A. Horger L, Harris DM, Berry A, Daroowalla F, Gillum S, Dil N, Cendan JC. Exploring students’ [pre-pandemic] use and the impact of commercial-off-the-shelf learning platforms on students’ national licensing exam performance: a focused review - BEME Guide No 72. Med Teach. 2022 Jul;44(7):707–719. Doi: 10.1080/0142159X.2022.2039380. 35271398

[17] Burk-Rafel J, Santen SA, Purkiss J. Study behaviors and USMLE step 1 performance: implications of a student self-directed parallel curriculum. Acad Med. 2017. 92:S67-74. 29065026 10.1097/ACM.0000000000001916

[18] Kortz MW, Kongs BM, Bisesi DR, Roffler M, Sheehy RM. A Retrospective and correlative analysis of academic and nonacademic predictors of COMLEX level 1 performance. J Osteopath Med. 2022 Jan 27;122(4):187–194. Doi: 10.1515/jom=2021-0175. 35084145

[19] Kind T, Olvet DM, Farina G, Kenda L, Sarandos SL, Yasunaga, Jokela JA, Simons RJ. Reading and study habits of medical students on clerkships and performance outcomes: a multi-institutional study. Med Sci Educ. 2021 Sep 29;31(6):1957–1966. Doi: 10.1007/s40670=021-021-01409-5. 34956707 PMC8651945

[20] Jeyaraju M, Linford H, Mendes TB, Caufield-Noll C; Tackett S. Factors leading to successful performance on U.S. National licensure exams for medical students: a scoping review. Acad Med. 2023 Jan 1;98(1):136–148. Doi: 10.1097/ACM.0000000000004877. 35857389

[21] Ball M, Lam L, Tigue M, Herzberg S, Finck L. The burden of unexpected costs in medical school. PLoS ONE 2024 Dec 5;19(12): e0312401. 10.1371/journal.pone.0312401 39637055 PMC11620594

[22] Farhan S, Kienzle D, Guler M, Siddique F, Fernandez A, Papanagnou D. The double-edged sword of third-party resources: examining use and financial burden of extracurricular tools in medical students. MedEdPublish (2016). 2025 Jan 2;14;4. https://doe.org/10.12588/mep.20120.2 10.12688/mep.20120.2 PMC11724203 39803636

[23] Mohri P, Koyluoglu YO, Seker ME, Sancak ŞN, Dikeç M, Mazlumoğlu AA, Gurol Y, Sungur IC. Medical students' perception of paid E-learning. PLoS ONE 2025 Mar 10;20(3):e0317340. 10.1371/journal.pone.0317340 40063656 PMC11892812

[24] Schultz M, Berryman DR. Collection practices for nontraditional online resources among academic health sciences libraries. J Med Libr Assoc. 2020 Apr;108(2):253–261. 10.5195/jmla.2020.791 32256236 PMC7069827

[25] Muellenbach JM, Robinson K, Fitterling L, Montgomery M, Bright HS. Textbook and board exam prep resource trends in academic health sciences libraries serving college of osteopathic medicine programs. J Electr Res Med Libr. 22 May 2024. Doi: 10.1080/15424065.2024.235111 PMC13367312 42453326

