# Supplementary material for: Board exam preparation resource trends in academic health sciences libraries serving colleges of osteopathic medicine programs
Source: J Med Libr Assoc. 2026 Jul 14;114(3):255–65. doi: 10.5195/jmla.2026.2320 (PMC13367312; doi:10.5195/jmla.2026.2320)
Supplement: Supplementary file 1 — Appendix A: Phase One Survey Questions [file jmla-114-3-255-s01.pdf]

College of Osteopathic Medicine (COM) Board Exam Preparation Resources Survey

Greetings!

You, as the director of a Health Sciences Library for a College of Osteopathic Medicine program in the United States (U.S.), are invited to participate in a research study entitled *Board Exam Preparation Resource Trends in Academic Health Sciences Libraries Serving Colleges of Osteopathic Medicine (COM) Programs*. The study, which is entirely voluntary, is being conducted by the following librarians: Joanne Muellenbach, California Health Sciences University (CHSU); Kyle Robinson, CHSU; Hsinliang (Oliver) Chen, Philadelphia College of Osteopathic Medicine; Lori Fitterling, Kansas City University; and Harold S. Bright IV, A.T. Still University.

The purpose of the study is to collect, analyze, and share data on the available board exam preparation resources in academic health sciences libraries that serve accredited COM programs. By completing and submitting this Qualtrics survey, you are indicating your consent to participate in the study. The estimated survey completion time is ten minutes, and all survey responses will be anonymous. If you have any questions regarding the survey or the research project in general, please contact the principal investigator, Joanne Muellenbach, at 559-549-6405, or jmuellenbach@chsu.edu.

****

Demographics

A. Please indicate the number of years you have served as an academic health sciences library director:

1-5 6-10 11-15 16+

B. Number of DO Students Served: < 500 501 – 1000 > 1000

Q1a. For each board exam prep resource listed below, please indicate if it is provided electronically, who subscribes, and which cohort(s) have access.

Resource & Link (if Available)	Electronic	Library Subscribes	Other Department Subscribes	OMS 1	OMS 2	OMS 3	OMS 4	Other OMS Cohorts (ex: Remediation)	Do not Know Cohort
AMBOSS (Physician Founder in Cologne, Germany)									
Blueprint/Med School Tutors (blueprintprep.com/medical/med-school/tutoring/meet-our-tutors)									
Boards and Beyond (McGraw-Hill)									
Kaplan - COMLEX Level 1 + Step 1 Qbank									
Kaplan - COMLEX-Level 2 + Step 2 Qbank									
First Aid Forward (McGraw-Hill)									
OnlineMedEd (OnlineMedEd.com)									
Osmosis (Elsevier)									
Pathoma USMLE Step 1 & Medical Course Review (Sattar)									
ScholarRx USMLE Study Tools (MedIQ Learning)									
TrueLearn - COMLEX Level 1									
TrueLearn - COMLEX Level 2									
TrueLearn - COMAT Shelf Exams									
TrueLearn - COMLEX Level 3									
UWorld - COMLEX Level 1									
UWorld - COMLEX Level 2									
UWorld - USMLE Step 1									
UWorld - USMLE Step 2									
If your library or other department is considering adding or dropping any board exam preparation resources, please elaborate in the space provided: [fill-in box to be provided]									

Q1b. If you have additional board exam prep resources not listed in Q1a, please list them below and indicate if they are provided electronically, who subscribes, and which cohort(s) have access.

Other Resources & Links (if Available)	Electronic	Library Subscribes	Other Department Subscribes	OMS 1	OMS 2	OMS 3	OMS 4	Other OMS Cohort (ex: Remediation)	Do Not Know Cohort
Other Resources & Links #1:									
Other Resources & Links #2:									
Other Resources & Links #3:									
Other Resources & Links #4:									
Other Resources & Links #5:									

Q2a. Below are *additional or more general* resources that may also include board exam preparation elements (e.g. Qbanks). Please indicate which of these are subscribed to by your library and indicate their format.

Resource & Link (if Available)	Print	Electronic	Both Print & Electronic
AccessMedicine (McGraw-Hill)			
Anki (apps.ankiweb.net)			
Board Review Series (LWW Health Library)			
BoardVitals (TetonData)			
Case Files Collection (McGraw-Hill)			
ClinicalKey (Elsevier)			
Doctors in Training (medicine.ac/category/usmle/dit/)			
Draw-It-To-Know-It (https://ditki.com)			
ExamMaster (www.exammaster.com)			
First Aid - Print Textbooks (McGraw-Hill)			
Goljan Audio Podcasts (www.podomatic.com/podcasts/goljanpathology)			
Master the Boards USMLE Step 1 (Kaplan)			
Master the Boards USMLE Step 2 CK (Kaplan)			
Master the Boards USMLE Step 3 (Kaplan)			
MedOne Education (Thieme)			

OMT Review (www.omtreview.com)	Print	Electronic	Both Print & Electronic
SecondLook (secondlook.med.umich.edu)			
SketchyMedical (www.sketchy.com)			
StatPearls (TetonData)			
Step Up to Medicine (LWW)			
If your library or other department is considering adding or dropping any of the additional board exam preparation resources, please elaborate in the space provided: [fill-in box to be provided].			

Q2b. If there are additional library resources to which your library subscribes that include board exam preparation elements or features, please list them below and indicate their format.

**Other Library Resources & Links (if Available)**	**Print**	**Electronic**	**Both Print & Electronic**
Other Resources & Links #1:			
Other Resources & Links #2:			
Other Resources & Links #3:			
Other Resources & Links #4:			
Other Resources & Links #5:			
Other Resources & Links #6:			

Q3. If your Library or other COM departments do NOT subscribe to board exam preparation resources, please select the statement that best explains why (*Select as many as apply*):

a. Not applicable / Our library or other COM department ***does*** subscribe to board exam prep resources.
b. Board exam prep resources are available on a private platform behind a firewall.
c. Board exam prep resources are developed in-house by our institution
d. Board exam prep resources are paid for by students directly
e. Board exam prep resources are paid for with dedicated funds controlled directly by the student
f. Board exam prep resources are not being used
g. Other: _______________

Q4a. Based on Q #1a-b and Q2a-b, what percentage of library collection budget dollars were allocated for print and electronic board exam preparation resources provided in the latest budget year?

Print % _____ + Electronic % ______ = Total 100%

Q4b. Are these percentages estimates?

Yes

No

Q5a. Please provide your annual COM library budget in the latest budget year for E-Resources:

a) E-resources: $0 - $250K; $250K – $500K; $500K - $750K; $750K – $1M; Over $1M;

Q5b. Please provide your annual COM library budget in the latest budget year for Print Resources:

b) Print resources: $0 - $49K; $50K - $99K; Over $100K

Q6. For COM board exam preparation resources in electronic format, please indicate if you or other COM departments participate in any of the following strategies for discounted pricing:

	Library	Other Department/Unit	N/A or Prefer not to Answer
h. Multi-year agreements (pay each year)			
i. Multi-year agreements (pay the total amount up front)			
j. Consortia			
i. If consortia, please list the consortia names			
k. Prefer not to answer			
l. Other:			

Q7. Please provide any library libguides or web page links to board exam preparation resources, texts, or software:

Link #1 Link #2 Link #3 Link #4 Link #5

Q8. **Would you be willing to be contacted by the Study Investigators for any follow-up? Yes No.** (if Yes, they will be taken to a new survey and will be asked to provide their name, institution, email and phone; if No, they will be taken to a new survey and will be asked to provide just their name and institution.
