# Supplementary material for: Board exam preparation resource trends in academic health sciences libraries serving colleges of osteopathic medicine programs
Source: J Med Libr Assoc. 2026 Jul 14;114(3):255–65. doi: 10.5195/jmla.2026.2320 (PMC13367312; doi:10.5195/jmla.2026.2320)
Supplement: Supplementary file 2 — Appendix B: Phase One Survey Demographic Questions [file jmla-114-3-255-s02.pdf]

Default Question Block

To ensure anonymity, demographic information is collected separately from the main survey. Your responses cannot be traced back to you or your previous answers."

I am willing to be contacted for a follow-up focus group interview to gather additional insights.

- ○ Yes
- ○ No

Please complete the following demographic information.

Name (First / Last)	
Institution Name	
Job Title	
Library	

Email

Phone Number

Please complete the following demographic information.

Name (First / Last)

Institution Name

Job Title

Library

Powered by Qualtrics
