# Supplementary material for: Board exam preparation resource trends in academic health sciences libraries serving colleges of osteopathic medicine programs
Source: J Med Libr Assoc. 2026 Jul 14;114(3):255–65. doi: 10.5195/jmla.2026.2320 (PMC13367312; doi:10.5195/jmla.2026.2320)
Supplement: Supplementary file 3 — Appendix C: Phase Two Interview Questions [file jmla-114-3-255-s03.pdf]

**Board Exam Prep Resource Trends Study - Focus Group Interviews**	**Introduction: "The Phase Two focus group interviews will be conducted via Zoom and should take no more than 30 minutes. The results will be anonymized, and all data will be handled with care to ensure confidentiality. Participation in the focus group interviews is completely voluntary and participants may choose not to answer a particular question at any time. By participating in the focus group interviews, participants understand that they are agreeing to participate in the study.**						

	Q1: what is your library's current decision-making process for selecting and providing access to board exam prep resources?	Q2. Has the library's processes as described in Q1 increased the value and visibility of the library, and if so, in what ways?	Q3. What do you think the library's role *should be* in the decision-making process for selecting and providing access to board exam prep resources?	Q4. How has your library's support for board prep resources evolved over the last few years?	Q5. How do you gather feedback about the board exam prep resources to which you subscribe?	Q6. Do you feel students use board prep resources more than traditional library resources, such as books, journals, or databases?	Q7. How has the availability of AI software played a part in supporting board exam prep?
